# Assessment of smartphone-based active distraction in association with audioanalgesia for overcoming airotor-related anxiety in children: a randomized controlled trial

**DOI:** 10.1186/s13104-025-07119-0

**Published:** 2025-01-30

**Authors:** Kritika Bali, Radhika Ailawadi, Y. M. Karuna, N. Srikant, Ashwin Rao, P. Anupama Nayak, Charisma Thimmaiah

**Affiliations:** 1 Manipal College of Dental Sciences, Mangalore, India; 2 Manipal Academy of Higher Education, Manipal, Karnataka India; 3 Department of Pediatric and Preventive Dentistry, Manipal College of Dental Sciences, Mangalore, India; 4 Department of Oral Pathology, Manipal College of Dental Sciences, Mangalore, India

**Keywords:** Behavior, Pediatric dentistry, Dental anxiety, Dental cavity preparations, Audio analgesia

## Abstract

**Background:**

Most children experience distress while visiting a dentist, above which the sound of the airotor and suction machine results in fear and difficulty in performing further procedures.

**Methods:**

This was a randomized controlled parallel-group study of 40 children aged 6–13 years who required cavity preparation via the airotor. The children were randomly allocated to either Group 1 (Piano music app; active distraction combined with audio analgesia) or Group 2 (basic behavioural guidance alone). Self-reported dental anxiety was measured via a modified child dental anxiety scale, and behavior was assessed via Venham’s and FLACC (Faces Legs Activity Cry and Consolability) scales. The data obtained were subjected to appropriate statistical analysis.

**Results:**

Self-reported dental anxiety was significantly lower in group 1 (*p* < 0.005). No significant difference between the groups was observed for the Venham and FLACC scores.

**Conclusion:**

Compared with basic behavioural guidance alone, the use of active distraction with audio analgesia in the form of the piano music app significantly decreased the degree of dental anxiety caused by the use of the airotor. This also resulted in clinically better cooperation by the child during cavity preparation.

**Trial Registration:**

Registered in the Clinical Trials Registry India (CTRI/2024/07/070160) dated 08/07/2024.

**Supplementary Information:**

The online version contains supplementary material available at 10.1186/s13104-025-07119-0.

## Introduction

The child’s first visit to the dentist is associated with fear of the unknown, followed by a feeling of anxiety. The literature has reported that the prevalence of dental anxiety is between 5.7% and 19.5% among pediatric dental patients [1]. Thus, in pediatric dentistry, dental anxiety is a concern, often resulting in numerous short-term consequences, such as increased stress, uncooperative behavior, heightened pain perception, avoidance of required treatment, and long-term consequences, such as persistent dental anxiety, avoidance of dental care into adulthood, increased risk of oral health issues, and generalized medical anxiety, potentially impacting overall well-being [2, 3].

Among the various reasons for dental fear and anxiety in children, the noise generated in a dental setup is ranked third. A study conducted by Muppa et al. [4] reported that the sound of the airotor was uncomfortable for the children included and that the noise generated significantly affected their dental avoidance behavior. An airotor is a high-speed dental handpiece that is regularly used for cavity/tooth preparation in day-to-day practice. The loud, high-pitched sound generated while an airotor is used is significant and often exceeds 66–80 dB [5]. It can be distressing to a pediatric patient, particularly if he/she already has dental anxiety, resulting in reduced cooperation. Other dental instruments, such as ultrasonic scalers, micro motors, and suction devices, also contribute to the noise generated in the dental clinic, further increasing anxiety in young patients. A study by Quisbati et al. [5] reported the generation of the highest noise levels in pediatric dental clinics.

Thus, to deliver high-quality dentistry to a child while also developing a positive attitude toward dental health, the dentist should have a good understanding of factors that might affect the behavior of children in a dental setting [6]. Distraction alters a child’s distress by disrupting attention and helping in the completion of dental procedures with minimal stress. There are two types of distraction techniques. In passive distraction, the child merely watches a video or listens to music without actually taking part in the activity. In contrast, in active distraction, the child actively takes part in activities that engage the child during the procedure. Playing with electronic devices is one such active distraction technique [7].

According to the prevailing evidence, active distraction is more efficient than passive distraction in overcoming dental pain and anxiety among pediatric dental patients [8]. Vishwakarma et al. [9] suggested that the Tell Play Do technique/mobile app is promising for lowering pediatric dental patients’ fear and anxiety, especially while using the airotor is effective at reducing children’s fear and anxiety of the associated dental procedures. Smartphone games were found to increase the adaptive behavior of children during dental procedures.

Another technique suggested for coping with pain and anxiety during dental treatments is audio analgesia [10]. It is said to minimize cardiovascular and neuroendocrine responses to the stress caused while performing various dental procedures [11]. To the best of our knowledge, there is no evidence regarding the effectiveness of the combination of active distraction and audio analgesia for reducing dental anxiety in pediatric patients. Thus, the present study was conducted to modify the airotor related anxiety of children by using smartphone-based artificial intelligence on the basis of the principles of active distraction and audio analgesia. The null hypothesis for the study was that there would be no difference in the airotor-related dental anxiety or behavior of the child when smartphone-based active distraction was used in combination with audio analgesia.

## Methods

This in vivo randomized controlled trial (CTRI no: CTRI/2024/07/070160) was conducted on outpatients who visited the Department of Pediatric and Preventive Dentistry. Ethical approval for the study was obtained from the Institutional Ethics Committee (Ref no: 23017). In addition, the study was conducted under the ethical standards of the 2013 amendment of the 1964 Helsinki Declaration.

Children aged 6–13 years who belonged to the positive (+) category of the Frankl behavior rating scale [12] when assessed during the initial dental visit and who required the use of an airotor for cavity preparations involving dentin in the anterior or posterior tooth were included in the study. The study procedure was explained to the parents, and written informed consent was obtained. When applicable, informed consent was also obtained from the child participants. Children for whom the dental procedure required the use of airotor, who followed the administration of local anaesthesia, who reported any systemic and/or mental illness, who had experienced previous use of airotor or whose parents did not sign the written informed consent form were excluded.

### Sample size calculation

On the basis of the values obtained in a study by Shekhar et al. [13], which used the following formula with 5% alpha error, 90% power of the study, and a clinically significant difference of 6 units, the required sample in each group was 20-.


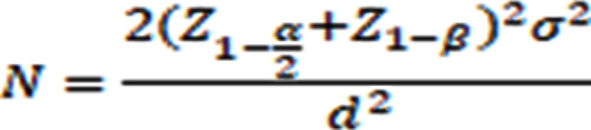


where.

alpha error = 5%.

Z(1-α/2): (Z score for the alpha error chosen) = 1.959963985.

1-beta (power) = 90%.

Z(1-β): (Z score for the power chosen) = 1.281551566.

σ1 = 7.2, σ2 = 4.4, σ = 5.8, d = 6.0.

Sample size = 19.637.

#### Intervention

Forty participants selected per the set inclusion and exclusion criteria were randomly allocated to one of the two study groups during their first indicated procedure for airotor usage.

#### Group 1:

**Fig. 1:**
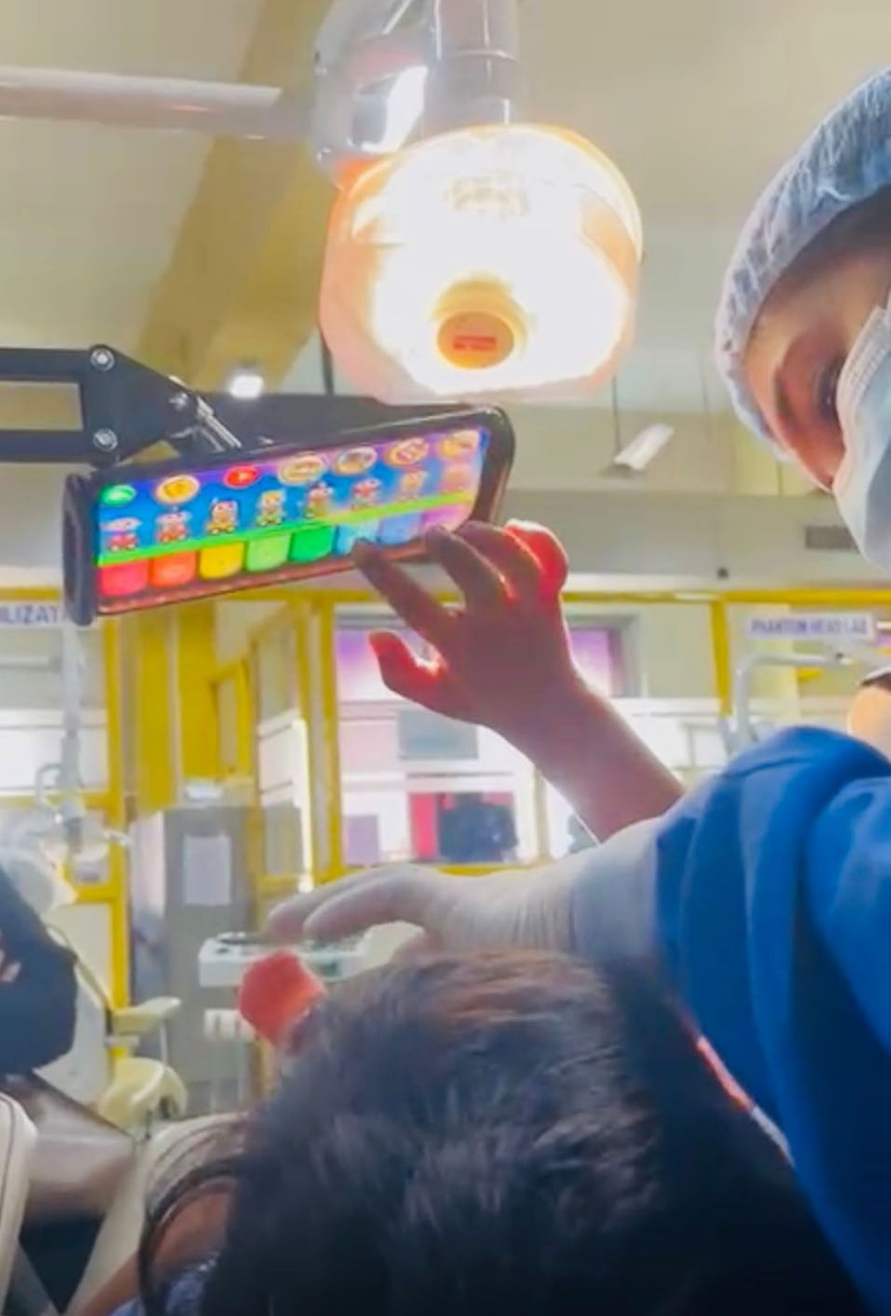
A smartphone-based app called Piano Music was used during the usage of the airotor for the indicated treatment; by tapping on the screen, the child played nursery rhymes

Piano music app (active distraction combined with audio analgesia): A smartphone-based app called Piano Music was used, in which the child could play nursery rhymes by tapping on the screen (Fig. 1). The game comes with a piano image on the screen and step-by-step instructions on playing the song. The phone was connected to a speaker via Bluetooth to enhance the sound effects.

#### Group 2:

Control group (basic behavior guidance techniques alone): Communication, which included a standard set of prescript verbal instructions about airotor usage for the indicated treatment in each patient with age-appropriate euphemisms (such as “I am going to clean your teeth using a small machine”). It might sound a bit like a buzzing noise and sprinkle water on your teeth. It may feel a little funny or ticklish. I expect you to open your mouth big and stay relaxed. In case at any point if you feel uncomfortable, please raise your hand and I will stop) was used, followed by verbal positive reinforcement (e.g., “You’re being so brave! I am truly proud that you are doing a great job, staying calm and relaxed.)

Communication and positive reinforcement were also used in Group 1, as in Group 2.

### Outcome measures

Demographic details such as name, age, sex, and past dental and medical experiences of the child were collected via a structured closed-ended questionnaire administered to the accompanying parent before the procedure. The modified Kuppuswamy scale was used to assess socioeconomic status [14]. The modified Corah’s dental anxiety scale (MDAS) was used to measure parental anxiety [15]. The temperament of the child was measured via the EAS (Emotionality, Activity, Shyness) Temperament Survey for Children (parental ratings) [16].

The dental anxiety of the child was recorded at two different time points: (1) before using the airotor and introducing the child to the intervention and (2) soon after completing the usage of the airotor, using the faces version of the Modified Child Anxiety Dental Scale (MCDAS(f)). It is a tool for measuring state anxiety that results from anticipation/uncertainty regarding a future threat [17]. The FLACC scale (Faces Legs Activity Cry and Consolability scale), which is scored by an observer, was used to assess the child’s behavior during the procedure. It is a valid behavioral scale that measures both psychological distress and pain in children [18]. The child’s behaviour during the entire duration of the procedure was also assessed via Venham’s behaviour rating scale. Venham’s behavior rating scale is the most helpful tool for evaluating the response of a child to dental stress. It captures a wide variety of manifestations of uncooperative behaviour in children ranging from zero (signifying total cooperation) to five (signifying general protest with no compliance or cooperation) [19]. The peak score observed was considered when the score was recorded [20]. The objective scoring of behavior was performed by two trained observers. Cohen’s kappa test was used to assess intraexaminer and interexaminer reliability.

#### Method of randomization and blinding:

Randomization was performed by a statistician who was blinded to the intervention. Computer-generated block sequences were used for randomization, followed by a random allocation of the samples to the blocks via a random number table. The treatment group codes were generated (A or B), and the sample numbers were entered into the cards, which were put inside sequentially numbered envelopes. The envelopes were covered with aluminum foil to enable allocation concealment and opened by the investigator just before initiating the intervention.

### Statistical analysis

The data collected from forty children in both groups were analysed via SPSS version 20.0 (IBM, Chicago, IL). The significance level was set at 5% (*p* < 0.05). The mean values of anxiety and behavior measures were obtained for each group. The Shapiro–Wilk test was used to assess the normality of the data. The chi-square test was used for the intergroup comparison of nonparametric data, and the independent t test was used for the comparison of parametric data. Multiple logistic regression was performed with the type of distraction, along with factors that influence dental anxiety, such as the socioeconomic status and gender of the child, parental anxiety, child temperament, and past medical and dental experiences of the child, as independent variables.

## Results

A comparison of the demographic details of the children included in the study revealed no statistically significant differences between the groups (Table 1). Similarly, when other possible confounding factors that may influence the dental anxiety of the child, such as temperament and parental anxiety, were compared, no significant difference was found (Table 2).

**Table 1:** Descriptive statistics of study subjects

Variable		Group 1 (Active distraction with audioanalgesia)	Group 2 (Control)	Chi-square	*P* value
Age (Mean ± SD)		8.4 ± 2.14	8.7 ± 2.56	-0.403^a^	0.689
Gender	Male	11 (55%)	9 (45%)	0.4	0.527
	Female	9 (45%)	11 (55%)		
Socioeconomic status	Lower Middle	5 (25%)	10 (50%)	3.429	0.33
	Upper Class	2 (10%)	1 (5%)		
	Upper Lower	1 (5%)	0 (0%)		
	Upper Middle	12 (60%)	9 (45%)		

^a^t value

**Table 2:** Comparison of temperament and MDAS scores between the groups using independent t test

Variable	Group 1 (Active distraction with audioanalgesia)	Group 2 (Control)	t	*P* value
Temperament (EAS)				
Emotionality	16.05 ± 4.98	14.75 ± 4.54	0.863	0.394
Activity	19 ± 4.51	19 ± 2.56	0	1
Shyness	12.25 ± 4.71	13.25 ± 4.2	-0.708	0.483
Parental anxiety (MDAS)	8.85 ± 4.42	12 ± 5.58	-1.979	0.055

### Dental anxiety of the child:

Among the six questions on the MCDAS(f) scale, having an injection in the gums and having the teeth removed were the most important anxiety-generating aspects for most of the children in both study groups (Supplementary table 1). 90% of the children in both groups were very anxious, fairly worried, worried a lot, and very worried before the intervention for both parameters. However, after the intervention, the combined percentage of the aforementioned scores in the intervention group remained at 90% and decreased to 80%, whereas in the control group, the percentage increased to 100% for both parameters. Before the intervention, the majority of the children in both groups responded slightly worried (40% and 45%, respectively), fairly worried (35% and 15%, respectively), or worried a lot (15% and 30%, respectively) about their teeth being filled, and the responses between the groups were not statistically significant (*p* = 0.41). On the other hand, after the intervention, 25% and 55% of the children in the intervention group were not worried/relaxed and slightly worried respectively, whereas 90% of the children in the control group were either fairly worried, or worried, or very worried, and this difference between the groups was statistically significant (*p* < 0.001). The differences in the mean scores before and after the intervention for each of the categories between the groups were statistically significant (Table 3). A p value of < 0.001 was found upon comparing the mean differences in the scores between the tested groups for completing the questionnaire and for the total MCDAS (f).

**Table 3:** Comparison of the mean difference in the MCDAS(f) scores obtained by subtracting the before-intervention scores from the after-intervention scores between the intervention and control groups

PARAMETER	Mean ± SD		t	*P* value
	Group 1 (Active distraction with Audioanalgesia) *N* = 20	Group 2 (Control) *N* = 20		
Going to the dentist generally	-0.25 ± 0.55^a^	0.25 ± 0.55	-2.874	0.007^*^
Having your teeth looked at	-0.3 ± 0.47^a^	0 ± 0.32	-2.349	0.025^*^
Having your teeth scraped or polished	-0.3 ± 0.57^a^	0.25 ± 0.44	-3.399	0.002^*^
Having an injection in your gums	-0.3 ± 0.47^a^	0.2 ± 0.41	-3.583	0.001^*^
Having a filling	-0.6 ± 0.5^a^	0.6 ± 0.68	-6.343	< 0.001^*^
Having teeth taken out	-0.4 ± 0.6^a^	0.3 ± 0.57	-3.785	0.001^*^
Total MCDAS(f)	-2.15 ± 1.5^a^	1.6 ± 1.23	-8.654	< 0.001^*^

^*^p < 0.05 is significant

^a^As the difference in the mean values was obtained by subtracting the before-intervention values from the after-intervention values, a negative value signifies a decrease in the anxiety scores.

### Children’s behavior during Airotor usage:

When children’s behavior during airotor usage was compared via Venham’s behavior rating scale, no significant difference was found between the groups (*P* = 0.158). 65% of Group 1 children extended total cooperation and best working conditions, with no crying against 40% of Group 2 children. Similarly, no statistically significant differences were found between the groups when FLACC scores were compared (*p* = 0.22). 45% of the children in the intervention group were relaxed and comfortable, whereas only 25% of the children in the control group were relaxed and comfortable (Table 4).

**Table 4:** Comparison of Venham’s behaviour rating scores and FLACC scores between the groups

	Score	*N*	Group 1 (Active distraction with audioanalgesia)	Group 2 (Control Group)	Chi-square	*P* value
Venham’sBehavior Rating Scale	0	21	13 (65%)	8 (40%)	5.19	0.158
	1	16	6 (30%)	10 (50%)		
	2	2	0 (0%)	2 (10%)		
	3	1	0 (0%)	1 (5%)		
FLACC score	0	14	9 (45%)	5 (25%)	7.01	0.22
	1–3	21	9 (45%)	12 (60%)		
	4–6	5	2 (10%)	3 (15%)		
	7–10	0	0 (0%)	0 (0%)		

## Discussion

The present study was conducted to help pediatric dental patients overcome airotor-generated dental anxiety. The Piano Music mobile application was used as the intervention in the study to reduce the dental anxiety of child patients during the use of the airotor for cavity preparation. In the piano music application, tapping the keys becomes a part of the play therapy and an active distraction method, whereas the rhyme that plays following tapping provides the effect of audio analgesia.

The results of the study revealed a significant reduction in the dental anxiety score (MCDAS(f)) after the intervention in the intervention group compared with the control group. These findings are supported by the study by Alsibai et al. [21], who concluded that active distraction in the form of video games using a wireless joystick displayed on a portable tablet on a dental chair was more helpful in reducing perceived pain and anxiety during dental procedures for school children. Similarly, a study by Guinot et al. [22] also used video games as an active distraction technique during pediatric restorative treatments and reported that active distraction was more efficient in lowering self-reported pain. However, the studies mentioned above compared the active distraction technique with the passive distraction technique. In contrast, our study employed the active distraction technique in combination with audioanalgesia in comparison to the basic behavioral techniques alone in the form of communication followed by positive reinforcement. Communication is a basic behavior management technique used universally in pediatric dentistry irrespective of the child’s coping threshold. Effective two-way communication with a pediatric patient, where the dentist empathetically speaks and patiently listens to the patient, is useful not only for establishing a rapport with the patient but also for reducing dental anxiety. When good behavior is shown by a child during treatment is followed by positive reinforcement, it further helps to reduce dental anxiety [23, 24].

The present study used the MCDAS(f), a self-reported anxiety scale, to evaluate the degree of dental anxiety experienced by the children. Lower MCDAS (f) scores were noted after the intervention in the intervention group. This may be attributed to the ability of the piano music app used in the study to actively engage the children during cavity preparation and the resulting music to compensate for the noise generated by the airotor, thus enabling the children to cope with their dental anxiety.

The scores recorded for a self-reported scale may be influenced by the cognitive ability of the child and other situational factors [13]. Thus, in addition to self-reported dental anxiety, we also evaluated the child’s behavior during the procedure via Venham’s behavior rating scale and FLACC scale.

The results of our study revealed that the majority of the children in the intervention group were relaxed, comfortable, and exhibited increased cooperation during cavity preparation, thus emphasizing the effectiveness of a coping mechanism in the form of a smartphone-based piano music application to overcome the resulting dental anxiety in children because the airotor generated noise. However, this study has a few limitations. This was a single-blind study. Only the statistician could be blinded due to the nature of the intervention. Additionally, this was a parallel-group study. The variability among the study participants and the resulting confounding factors is the drawback of the parallel design [25]. We chose a parallel group study design with a crossover design to prevent the possible influence of earlier appointments on upcoming dental experiences, namely, the spillover effect [26]. Confounding factors such as demographic details, the temperament of the child, and parental anxiety were recorded and analysed. No significant differences were noted between the groups, thus ruling out their possible effect on the study outcome.

## Conclusion

Within the study’s limitations, it can be concluded that, for Frankl’s study, in the positive (+) category of 6- to 13-year-old children, a combination of active distraction and audio analgesia delivered in the form of a piano music app helped reduce dental anxiety during cavity preparation performed via an airotor. Compared with the basic behavior management technique, this approach results in more relaxed and comfortable children who achieve better cooperation during cavity preparation.

Given the positive impact observed, we recommend integrating such nonpharmacological behavioral interventions into routine pediatric dental care, especially for patients who are anxious or uncooperative. However, further research is necessary to explore the long-term effects of music-based active distraction interventions, assess their efficacy across different age groups and anxiety levels, and compare their impact with that of passive distraction techniques. Thus, future studies with larger sample sizes and diverse populations will help validate these findings and refine strategies for optimizing pediatric dental care.

## Electronic supplementary material

Below is the link to the electronic supplementary material.

Supplementary Material 1

Supplementary Material 2

## Data Availability

The raw data used in the study are stored locally with the corresponding author and are freely available upon request.
