# Supplementary material for: Assessment of smartphone-based active distraction in association with audioanalgesia for overcoming airotor-related anxiety in children: a randomized controlled trial
Source: BMC Res Notes. 2025 Jan 30;18:46. doi: 10.1186/s13104-025-07119-0 (PMC11783720; doi:10.1186/s13104-025-07119-0)
Supplement: Supplementary file 1 — Supplementary Material 1 [file 13104_2025_7119_MOESM1_ESM.docx]

| **Supplemental Table: Frequency distribution of the study subjects to different score categories of MCDAS(f) as assessed before and after the intervention.** | | | | | | | |
| --- | --- | --- | --- | --- | --- | --- | --- |
| **Time-point of assessment with respect to the intervention** | **Parameter assessed** | **Scores** | **N** | **Group 1 (N (%))** | **Group 2 (N (%))** | **Chi-square** | **P value** |
| Before | Going to the dentist generally | Not Anxious | 24 | 10 (50) | 14 (70) | 3.744 | 0.291 |
|  |  | Slightly Anxious | 13 | 7 (35) | 6 (30) |  |  |
|  |  | Very Anxious | 1 | 1 (5) | 0 (0) |  |  |
|  |  | Extremely Anxious | 2 | 2 (10) | 0 (0) |  |  |
| After |  | Not Anxious | 21 | 11 (55) | 10 (50) | 3.298 | 0.348 |
|  |  | Slightly Anxious | 16 | 7 (35) | 9 (45) |  |  |
|  |  | Fairly Anxious | 1 | 0 (0) | 1 (5) |  |  |
|  |  | Very Anxious | 2 | 2 (10) | 0 (0) |  |  |
| Before | Having your teeth looked at | Not Anxious | 19 | 9 (45) | 10 (50) | 2.105 | 0.349 |
|  |  | Slightly Anxious | 19 | 9 (45) | 10 (50) |  |  |
|  |  | Fairly Anxious | 2 | 2 (10) | 0 (0) |  |  |
| After |  | Not Anxious | 23 | 13 (65) | 10 (50) | 0.921 | 0.337 |
|  |  | Slightly Anxious | 17 | 7 (35) | 10 (50) |  |  |
| Before | Having your teeth scraped or polished | Not Anxious | 10 | 5 (25) | 5 (25) | 5.087 | 0.278 |
|  |  | Slightly Anxious | 23 | 9 (45) | 14 (70) |  |  |
|  |  | Fairly Anxious | 4 | 3 (15) | 1 (5) |  |  |
|  |  | Very Anxious | 2 | 2 (10) | 0 (0) |  |  |
|  |  | Extremely Anxious | 1 | 1 (5) | 0 (0) |  |  |
| After |  | Not Anxious | 8 | 6 (30) | 2 (10) | 4 | 0.261 |
|  |  | Slightly Anxious | 25 | 10 (50) | 15 (75) |  |  |
|  |  | Fairly Anxious | 6 | 3 (15) | 3 (15) |  |  |
|  |  | Very Anxious | 1 | 1 (5) | 0 (0) |  |  |
| Before | Having an injection in your gums | Slightly Anxious | 2 | 1 (5) | 1 (5) | 2.716 | 0.438 |
|  |  | Fairly Anxious | 9 | 6 (30) | 3 (15) |  |  |
|  |  | Very Anxious | 19 | 7 (35) | 12 (60) |  |  |
|  |  | Extremely Anxious | 10 | 6 (30) | 4 (20) |  |  |
| After |  | Slightly Anxious | 2 | 2 (10) | 0 (0) | 6.143 | 0.105 |
|  |  | Fairly Anxious | 11 | 8 (40) | 3 (15) |  |  |
|  |  | Very Anxious | 17 | 6 (30) | 11 (55) |  |  |
|  |  | Extremely Anxious | 10 | 4 (20) | 6 (30) |  |  |
| Before | Having a filling | Not Anxious | 3 | 2 (10) | 1 (5) | 3.992 | 0.407 |
|  |  | Slightly Anxious | 17 | 8 (40) | 9 (45) |  |  |
|  |  | Fairly Anxious | 10 | 7 (35) | 3 (15) |  |  |
|  |  | Very Anxious | 9 | 3 (15) | 6 (30) |  |  |
|  |  | Extremely Anxious | 1 | 0 (0) | 1 (5) |  |  |
| After |  | Not Anxious | 6 | 5 (25) | 1 (5) | 22.333 | **<0.001** |
|  |  | Slightly Anxious | 12 | 11 (55) | 1 (5) |  |  |
|  |  | Fairly Anxious | 12 | 4 (20) | 8 (40) |  |  |
|  |  | Very Anxious | 8 | 0 (0) | 8 (40) |  |  |
|  |  | Extremely Anxious | 2 | 0 (0) | 2 (10) |  |  |
| Before | Having teeth taken out | Not Anxious | 1 | 1 (5) | 0 (0) | 2.792 | 0.593 |
|  |  | Slightly Anxious | 3 | 1 (5) | 2 (10) |  |  |
|  |  | Fairly Anxious | 9 | 6 (30) | 3 (15) |  |  |
|  |  | Very Anxious | 17 | 8 (40) | 9 (45) |  |  |
|  |  | Extremely Anxious | 10 | 4 (20) | 6 (30) |  |  |
| After |  | Not Anxious | 2 | 2 (10) | 0 (0) | 12.7 | **0.013** |
|  |  | Slightly Anxious | 2 | 2 (10) | 0 (0) |  |  |
|  |  | Fairly Anxious | 8 | 5 (25) | 3 (15) |  |  |
|  |  | Very Anxious | 20 | 11 (55) | 9 (45) |  |  |
|  |  | Extremely Anxious | 8 | 0 (0) | 8 (40) |  |  |
